# Combination of static and dynamic neural imaging features to distinguish sensorineural hearing loss: a machine learning study

**DOI:** 10.3389/fnins.2024.1402039

**Published:** 2024-06-12

**Authors:** Yuanqing Wu, Jun Yao, Xiao-Min Xu, Lei-Lei Zhou, Richard Salvi, Shaohua Ding, Xia Gao

**Affiliations:** ^1^Department of Otorhinolaryngology Head and Neck Surgery, Nanjing Drum Tower Hospital Clinical College of Nanjing Medical University, Nanjing, China; ^2^Department of Otorhinolaryngology Head and Neck Surgery, Nanjing First Hospital, Nanjing Medical University, Nanjing, China; ^3^Department of Radiology, Nanjing First Hospital, Nanjing Medical University, Nanjing, China; ^4^Center for Hearing and Deafness, University at Buffalo, The State University of New York, Buffalo, NY, United States; ^5^Department of Radiology, The Affiliated Taizhou People's Hospital of Nanjing Medical University, Taizhou School of Clinical Medicine, Nanjing Medical University, Taizhou, China

**Keywords:** sensorineural hearing loss, functional imaging, static features, dynamic features, machine learning

## Abstract

**Purpose:**

Sensorineural hearing loss (SNHL) is the most common form of sensory deprivation and is often unrecognized by patients, inducing not only auditory but also nonauditory symptoms. Data-driven classifier modeling with the combination of neural static and dynamic imaging features could be effectively used to classify SNHL individuals and healthy controls (HCs).

**Methods:**

We conducted hearing evaluation, neurological scale tests and resting-state MRI on 110 SNHL patients and 106 HCs. A total of 1,267 static and dynamic imaging characteristics were extracted from MRI data, and three methods of feature selection were computed, including the Spearman rank correlation test, least absolute shrinkage and selection operator (LASSO) and t test as well as LASSO. Linear, polynomial, radial basis functional kernel (RBF) and sigmoid support vector machine (SVM) models were chosen as the classifiers with fivefold cross-validation. The receiver operating characteristic curve, area under the curve (AUC), sensitivity, specificity and accuracy were calculated for each model.

**Results:**

SNHL subjects had higher hearing thresholds in each frequency, as well as worse performance in cognitive and emotional evaluations, than HCs. After comparison, the selected brain regions using LASSO based on static and dynamic features were consistent with the between-group analysis, including auditory and nonauditory areas. The subsequent AUCs of the four SVM models (linear, polynomial, RBF and sigmoid) were as follows: 0.8075, 0.7340, 0.8462 and 0.8562. The RBF and sigmoid SVM had relatively higher accuracy, sensitivity and specificity.

**Conclusion:**

Our research raised attention to static and dynamic alterations underlying hearing deprivation. Machine learning-based models may provide several useful biomarkers for the classification and diagnosis of SNHL.

## Introduction

Sensorineural hearing loss (SNHL) is a global public health problem and is often unrecognized by patients (Tordrup et al., 2022). It is estimated that SNHL currently affects 1.5 billion people worldwide and will affect 9 billion people by the year 2050 (Disease et al., 2018). SNHL can detract from quality of life at the individual level and cause a severe economic burden at the societal level (Collaborators, 2021). Known consequences of SNHL include not only hearing and communication difficulties but also social isolation, anxiety, depression and cognitive impairments (Olusanya et al., 2019), indicating the key role of neural substrates.

Neural imaging is a powerful tool and significantly enhances our knowledge about the human brain. Previous studies mostly used resting-state MRI to study SNHL, including fractional amplitude of low-frequency fluctuation (fALFF), regional homogeneity (ReHo) and degree centrality (DC). fALFF can suppress physiological noise (such as the vicinity of large blood vessels, cisterns and ventricles) and measure the contribution of low-frequency fluctuation within specific frequency bands (Zou et al., 2008). Slow 5 (0.01–0.027 Hz) and slow 4 (0.027–0.073 Hz) have better sensitivity to gray matter (Xu et al., 2019b). ReHo is calculated on Kendall coefficient consistency and defined as the similarity or nonparametric concordance of adjacent voxel time series (Li et al., 2013). Additionally, DC describes functional integration among the whole brain using graph theoretic techniques (Guan et al., 2022). These characteristics are relatively static and treat the spatial and temporal information of the functional brain as separate parts.

Emerging evidence suggests that the human brain is a complex dynamic system that is interconnected across time and space (Deco et al., 2011; Hou et al., 2018; Kong et al., 2023). Multilayer network analysis with the use of sliding windows provides the opportunity to detect the dynamic network configuration over time-resolved fMRI (Pedersen et al., 2018). The vital feature is the network switching rate or node flexibility, which is defined as the percentage of time when a node transitions to different functional networks (Bassett et al., 2011). However, the sliding windows and the length of step require prespecification and are correlated with global synchronization and temporal stability (Hutchison et al., 2013). Another analysis with the hidden Markov model (HMM) can overcome the above limitation and discrete brain states in a data-driven manner, describing the transition probabilities between states (Stevner et al., 2019). Multilayer network analysis and HMM analysis have been computed in some neuropsychological diseases, such as depression, schizophrenia and dementia (Wang et al., 2020, 2021; Li et al., 2021). To our knowledge, no study has examined the dynamic characteristics of SNHL using these processing methods.

Many studies have used traditional methodologies to diagnose SNHL presence with the help of clinical doctors, while machine learning has been widely applied to automatically identify various datasets and risk factors for diseases. Existing studies conducted machine learning models with hearing thresholds and RNA expression to diagnose hereditary hearing loss (Luo et al., 2021), noise-induced hearing loss (Chen et al., 2021) and SNHL (Shew et al., 2019), but they ignored the involvement of neural functions. fMRI-based radiomics can be utilized to explore neurological disease biomarkers and underlying mechanisms, such as cognitive impairments and depression (Shi et al., 2021; Shin et al., 2021; Chand et al., 2022).

In the present study, our hypothesis is machine learning models with a combination of static and dynamic brain feature can efficiently distinguish SNHL patients and controls. These machine learning models were applied to deidentified datasets and used to calculate the presence of SNHL. Moreover, to our knowledge, our research might be the early one to apply multi-order radiomics in identifing SNHL biomarkers, and this approach would contribute to a better understanding of machine learning tools to predict susceptibility to SNHL.

## Materials and methods

### Participants and ethics statement

A total of 110 bilateral SNHL patients and 106 age-and sex-matched healthy controls (HCs) were recruited from the Otolaryngology Department of Nanjing First Hospital and the local community via advertisements. This study was conducted with approval from the Research Ethics Committee of our hospital, and written informed consent was obtained from each participant prior to study participation. A pure tone audiometry (PTA) test was computed to evaluate the hearing threshold and the diagnosis of SNHL. A tympanometry test was conducted to confirm the function of the middle ear. The Mini-Mental State Examination (MMSE), Montreal Cognitive Assessment (MoCA), verbal fluency test (VFT), Trail Making Test-Part A/B (TMT-A/B), auditory verbal learning test (AVLT), clock drawing test (CDT), digit span test (DST), digit symbol substitution test (DSST), self-rating anxiety scale (SAS), and Hamilton Depression Scale (HAMD) were used to evaluate cognition and mental condition. We excluded individuals if they (1) suffered from pulsatile tinnitus, hyperacusis, Meniere’s disease, conductive deafness, Parkinson’s disease, Alzheimer’s disease and major illnesses; (2) had a history of brain injury, drug addiction, smoking or alcohol addiction; or (3) had MRI contraindications.

### Imaging acquisition and data preprocessing

A 3.0 Tesla MRI with an 8-channel head coil (Ingenia, Philips Medical Systems, Netherlands) was used for the imaging acquisition. All subjects were instructed to remain awake and avoid thinking about special things during the scanning. Foam padding was used to minimize head motion, and earplugs were used to attenuate scanning noise (about 32 dB). Structural images were acquired using a 3D-T1 sequence, and the parameters were as follows: repetition time (TR) = 8.1 ms, echo time (TE) = 3.7 ms, slices = 170, thickness = 1 mm, gap = 0 mm, flip angle (FA) = 8°, field of view (FOV) = 256 mm × 256 mm, matrix = 256 × 256. Functional images were acquired using a gradient echo-planar imaging sequence as follows: time points = 240, TR = 2000 ms, TE = 30 ms, slice = 36, thickness = 4 mm, gap = 0 mm, FA = 90°, FOV = 240 mm × 240 mm, matrix = 60 × 60.

**Figure 1:**
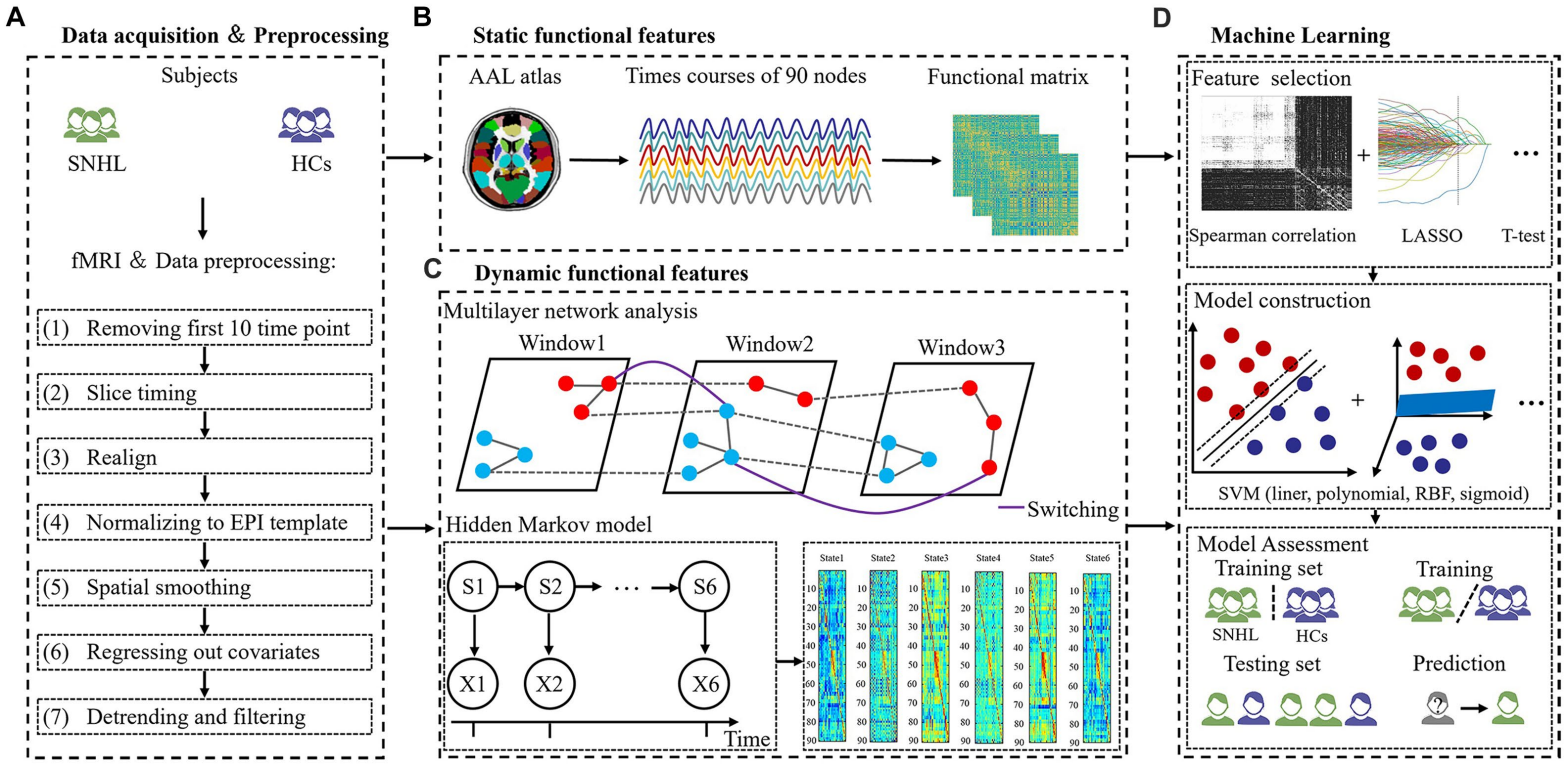
The flowchart of the experiment. **(A)** Data acquisition and processing. **(B)** Static functional features. **(C)** Dynamic functional features. **(D)** Machine learning.

Using tools in the Graph Theoretical Network Analysis Toolbox for Imaging Connectomics (GRETNA),^1^ functional MRI were preprocessed following the pipelines: (1) removing the first 10 time points for signal equilibrium, the remaining 230 images underwent subsequent analysis, (2) slice timing, (3) realign, (4) normalizing to EPI template (reslicing voxel size as 3 × 3 mm^3^), (5) spatial smoothing with a 6 mm full width at half maximum (FWHM), (6) regressing out covariates using Friston-24 parameters, and (7) detrending and filtering with a band from 0.01 to 0.1 Hz (Figure 1A). Subjects with head motion >2.0 mm or rotation angle >2.0° in any direction were removed from analysis, and nobody was excluded in our study.

### Structural data analysis

The 3D-T1 sequence was used for structural analysis using DARTEL voxel-based morphometry (VBM) method according to previous study (Lin et al., 2013). The analysis steps were as follows: (1) segmenting T1 images into gray matter (GM), white matter and cerebrospinal fluid, (2) constructing GM templates from the dataset; (3) performing non-linear warping of segmented images; (4) spatial normalization; (5) smoothing with a 6 mm FWHM. The VBM analysis demonstrated that SNHL was not related to significant structural alterations in present study.

### Static and dynamic analysis

Static brain characteristics included f1ALFF (0.01–0.027 Hz), f2ALFF (0.027–0.073 Hz), ReHo, binary DC (BDC) and weighted DC (WDC), which is consistent with previous studies (Xu et al., 2019b; Zeng et al., 2021). Then, we extracted representative signals of 90 nodes based on the anatomical automatic labeling (AAL) atlas using REST software^2^ (Figure 1B).

To investigate the dynamic brain features, we applied a multilayer network and HMM to time courses that were extracted from 90 nodes. We computed a sliding window method to calculate a dynamic function where the window size was set to 40 and the overlap was set to 0.975. The ordinal GenLouvain algorithm was used to track switching rates (SR)/node flexibilities (NF), and detailed information was similar to prior studies (Pedersen et al., 2018; Yang et al., 2020). This model is governed by γ and ω parameters, and we used a range of parameters, including γ = [0.9, 1, 1.1] and ω = [0.5, 0.75, 1], in the present study.

Additionally, we also applied HMM with a multivariate Gaussian observation model. According to an existing paper, the number of states assumed for the signal dynamics was specified as 6 (Moretto et al., 2022). Three global temporal characterizations of HMM dynamics were estimated from time courses, including fractional occupancies (FO) and SR (Lin et al., 2022). FO is defined as the ratio of activated HMM states across the all-time course. SR is the frequency of transitions between different states (Figure 1C).

### Feature extraction

All features were based on the AAL 90 atlas in our study. Static features include f1ALFF (1–90), f2ALFF (1–90), ReHo (1–90), BDC (1–90) and WDC (1–90) [90 × 5 = 450 features]. Dynamic brain features include NF 1 → 1 (1–90), NF 1 → 2 (1–90), NF 1 → 3 (1–90), NF 2 → 1 (1–90), NF 2 → 2 (1–90), NF 2 → 3 (1–90), NF 3 → 1 (1–90), NF 3 → 2 (1–90), NF 3 → 3 (1–90) using multilayer network analysis, as well as FO (1–6) and SR using the HMM method [90 × 9 + 6 + 1 = 817 features] (Figure 1D).

### Feature selection

The dimension of 1,267 extracted features is relatively high; therefore, so we needed to perform some dimensionality reduction on these features, as follows: (1) Spearman rank correlation test (the top 1% features were reserved), (2) least absolute shrinkage and selection operator (LASSO) with correlation coefficients at lambda = 100 and alpha = 1, and (3) t test and LASSO (features with a *p*-value <0.05 with independent sample t test were retained to perform LASSO subsequently). The above three strategies were computed with 5-fold cross-validation.

### Model construction and assessment

For model construction, four types of support vector machine (SVM) models were chosen as the classifiers, including linear, polynomial, radial basis functional kernel (RBF) and sigmoid SVM (Shin et al., 2021). We compared the ability of the models to distinguish between SNHL and HC using the following indicators: the receiver operating characteristic curve (ROC), area under the curve (AUC), sensitivity, specificity and accuracy. To estimate the generalizability and transportability of machine learning models, 5,000 permutation tests were performed.

## Results

### Demographic characteristics and clinical data

Detailed information on demographic characteristics and clinical data is summarized in Table 1. A total of 110 SNHL patients and 106 HCs were matched in terms of sex, age and education duration (*p* > 0.05). The hearing thresholds of each ear at 0.25, 0.5, 1, 2, 4, and 8 kHz in the SNHL group were significantly higher than those in the HC group (*p* < 0.001). The SNHL group performed worse on the CFT-delay and TMT-A/B tests, which are associated with visual memory recall and cognitive flexibility, respectively. Moreover, the SAS and HAMD scores of patients with SNHL were higher than those of HCs.

**Table 1:** Demographic information and clinical characteristics.

	SNHL	HCs	*p*-value
*Demographic information*			
Number of subjects	110	106	–
Gender (Male/Female)	63/47	70/36	0.186
Age (years)	57.47 ± 7.65	56.53 ± 7.16	0.350
Education (years)	11.78 ± 2.28	11.42 ± 2.08	0.229
*Neuropsychological tests*			
MMSE	28.87 ± 0.97	29.06 ± 1.13	0.200
MoCA	27.06 ± 1.73	26.89 ± 1.68	0.447
VFT	14.37 ± 3.93	15.23 ± 3.78	0.101
CFT	29.97 ± 15.96	32.66 ± 6.82	0.107
CFT-delay	17.46 ± 4.35	24.82 ± 9.08	<0.001***
TMT-A	81.01 ± 44.50	70.16 ± 24.25	0.027*
TMT-B	183.85 ± 67.83	155.92 ± 63.95	0.002**
AVLT	35.13 ± 7.72	34.64 ± 7.77	0.645
AVLT-delay	6.92 ± 2.37	6.74 ± 2.36	0.572
CDT	3.55 ± 0.53	3.48 ± 0.56	0.324
DST	11.43 ± 1.92	11.55 ± 1.84	0.640
DSST	69.38 ± 9.02	69.06 ± 9.02	0.791
SAS	37.37 ± 7.83	33.38 ± 4.97	<0.001***
HAMD	5.62 ± 3.49	4.71 ± 3.04	0.042*
*Hearing thresholds of each ear*			
Right-0.25 kHz	37.09 ± 25.53	18.07 ± 4.76	<0.001***
Right-0.5 kHz	36.95 ± 28.93	14.58 ± 5.94	<0.001***
Right-1 kHz	42.50 ± 31.06	17.08 ± 7.23	<0.001***
Right-2 kHz	44.48 ± 31.86	17.41 ± 7.75	<0.001***
Right-4 kHz	52.95 ± 28.22	17.78 ± 7.78	<0.001***
Right-8 kHz	58.55 ± 25.39	20.24 ± 11.37	<0.001***
Left-0.25 kHz	34.82 ± 24.84	15.00 ± 5.35	<0.001***
Left-0.5 kHz	36.91 ± 28.23	14.29 ± 4.50	<0.001***
Left-1 kHz	39.81 ± 28.96	15.66 ± 5.82	<0.001***
Left-2 kHz	42.89 ± 28.81	15.24 ± 6.98	<0.001***
Left-4 kHz	51.36 ± 26.06	14.95 ± 7.91	<0.001***
Left-8 kHz	55.86 ± 26.78	18.82 ± 9.17	<0.001***

Data are expressed as Mean ± SD.

*p*-values were calculated with the independent *t*-test or *x*^2^ test, as appropriate.

*p < 0.05, **p < 0.01, ***p < 0.001.

SNHL, sensorineural hearing loss; HCs, healthy controls; MMSE, Mini-Mental State Exam; MoCA, Montreal Cognitive Assessment; VFT, Verbal Fluency Test; TMT-A, Trail Making Test-Part A; TMT-B, Trail Making Test-Part B; AVLT, Auditory Verbal Learning Test; CDT, Clock Drawing Test; DST, Digit Span Test; DSST, Digit Symbol Substitution Test; SAS, Self-rating Anxiety Scale; HAMD, Hamilton Depression Scale.

### Feature selection

**Figure 2:**
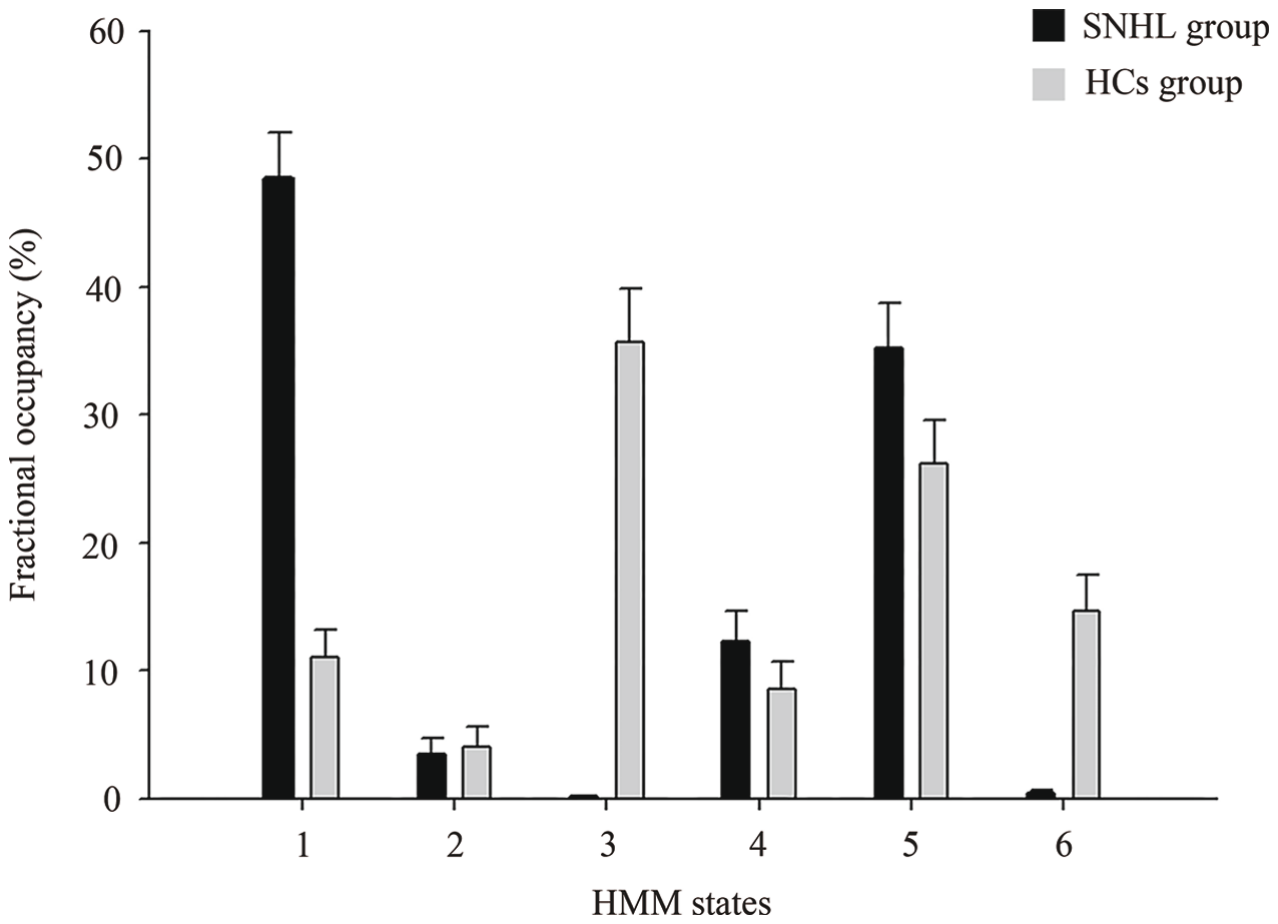
Alterations in fractional occupancies of each state between SNHL and HCs groups using HMM. HMM, hidden Markov model; SNHL, sensorineural hearing loss; HCs, healthy controls.

In the dynamic analysis with the HMM model, we chose six states, and the HMM inference estimated the time course of each state. As shown in Figure 2, the FO of HMM state 1 with SNHL was significantly increased (*p* < 0.001), and the FO of HMM states 3 and 6 with SNHL was significantly decreased (*p* < 0.001). No significance was observed in other states. In addition, the SR of SNHL patients (mean ± SD, 0.08 ± 0.05) was higher than that of HCs (mean ± SD, 0.06 ± 0.05) (*p* = 0.001), indicating a special pattern of temporal configuration in patients following bilateral hearing deprivation.

**Figure 3:**
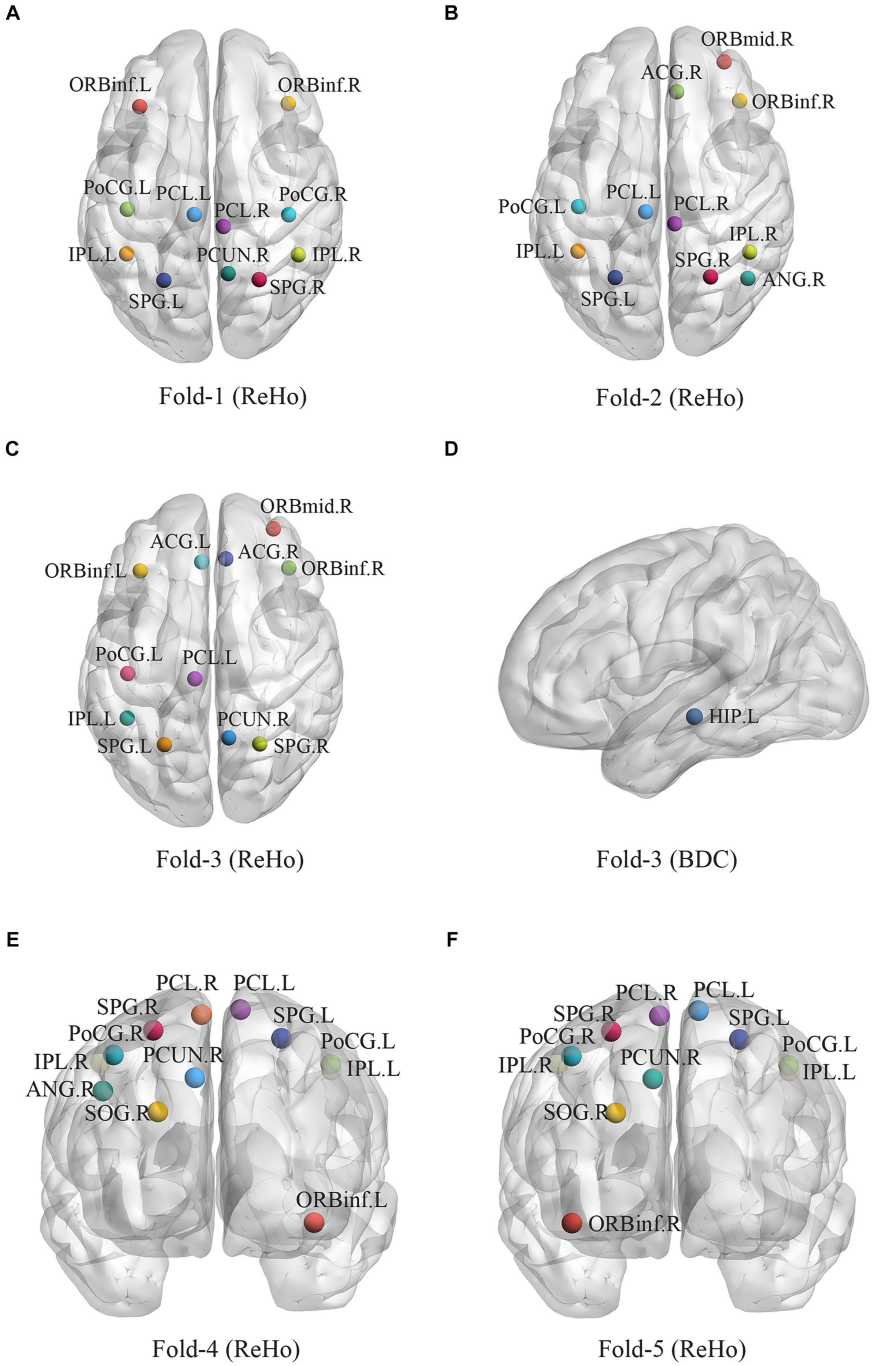
Selected static and dynamic features using spearman in five-fold cross-validation. **(A)** Fold-1 in ReHo; **(B)** Fold-2 in ReHo; **(C)** Fold-3 in ReHo; **(D)** Fold-3 in BDC; **(E)** Fold-4 in ReHo; **(F)** Fold-5 in ReHo. ORBinf, inferior frontal gyrus, orbital part; ORBmid, middle frontal gyrus, orbital part; ACG, anterior cingulate gyrus; PoCG, postcentral gyrus; PCL, paracentral lobule; IPL, inferior parietal lobule, PCUN, precuneus; SPG, superior parietal gyrus; ANG, angular gyrus; SOG, superior occipital gyrus; ANG, angular gyrus; HIP, hippocampus; ReHo, regional homogeneity; BDC,binary degree centrality.

Among 1,267 imaging features, we computed three methods of feature selection. First, the discriminative regions using Spearman rank correlation in fivefold cross-validation are shown in Figure 3 and Supplementary Table S1, including FO1, FO2, FO4, BDC of the hippocampus (HIP) and ReHo values in the inferior frontal gyrus, orbital part, middle frontal gyrus, orbital part, anterior cingulate gyrus, postcentral gyrus paracentral lobule, inferior parietal lobule, precuneus, superior parietal gyrus, angular gyrus and superior occipital gyrus. Second, selected features using LASSO are summarized in Supplementary Figure S1 and Supplementary Table S2, as static characteristics (including f1ALFF, f2ALFF, ReHo, BDC and WDC) and dynamic features (including FO and NF) were involved here. Third, we applied a t test as well as LASSO to select features, and only dynamic characteristics (NF) were recruited (Supplementary Figure S2 and Supplementary Table S3).

### Performance of the classification for SNHL

**Figure 4:**
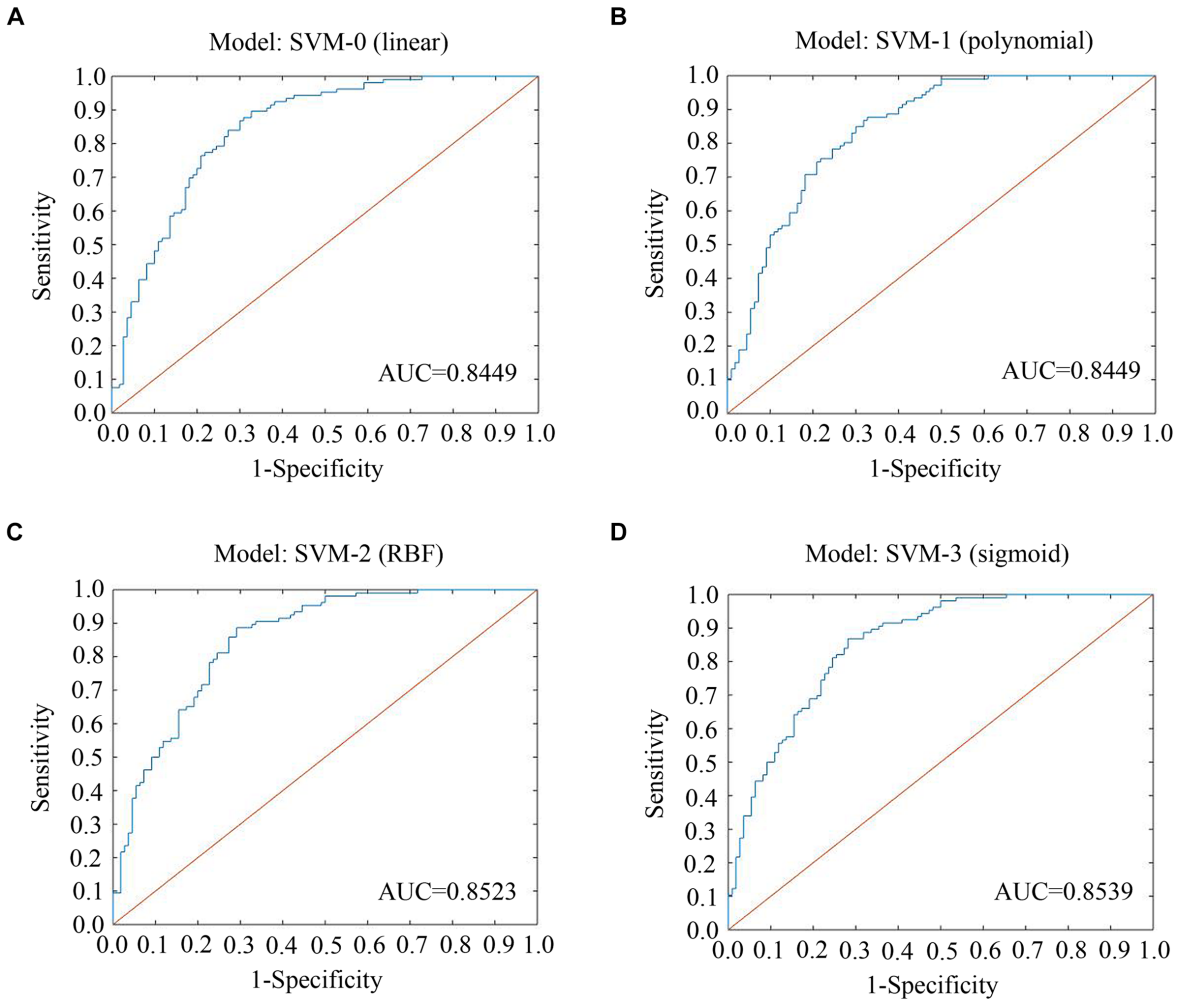
ROC curves and AUCs of the four SVMs using features selected by spearman. **(A)** Model: SVM-0; **(B)** Model: SVM-1; **(C)** Model: SVM-2; **(D)** Model: SVM-3. ROC, receiver operating characteristic curve; AUC, area under the curve; SVM, support vector machine; LASSO, least absolute shrinkage and selection operator; RBF, radial basis functional kernel.

The ROCs and their AUCs were calculated to compare the classification performance among the four machine learning models. Different SVM models (linear, polynomial, RBF and sigmoid) using selected features by Spearman rank correlation achieved a performance with AUCs of 0.8449, 0.8449, 0.8523, and 0.8539, respectively (Figure 4). Based on static and dynamic features from LASSO analysis, the AUCs of the four SVM models (linear, polynomial, RBF and sigmoid) were 0.8075, 0.7340, 0.8462, and 0.8562, respectively (Supplementary Figure S3). Classification of the results using linear, polynomial, RBF and sigmoid SVM after t test and LASSO analysis were 0.7982, 0.7033, 0.8442, and 0.8412, respectively (Supplementary Figure S4). Detailed information about the performance of the four SVMs based on the three methods of feature selection is shown in Table 2, including AUC, accuracy, sensitivity and specificity. The RBF SVM and sigmoid SVM exhibited relatively high sensitivity and specificity of classification for SNHL.

**Table 2:** Mean value of 5-fold cross-validation using four machine learning algorithms after different methods of feature selection.

Feature selection	Classifier	AUC	Accuracy	Sensitivity	Specificity
Spearman	SVM-0	0.8449	0.7683	0.7545	0.7830
Spearman	SVM-1	0.8449	0.7223	0.8545	0.5849
Spearman	SVM-2	0.8523	0.7820	0.7545	0.8113
Spearman	SVM-3	0.8539	0.7774	0.7455	0.8113
Lasso	SVM-0	0.8075	0.7267	0.6909	0.7642
Lasso	SVM-1	0.734	0.5193	1	0
Lasso	SVM-2	0.8462	0.7821	0.7091	0.8585
Lasso	SVM-3	0.8562	0.7682	0.6636	0.8774
T + Lasso	SVM-0	0.7982	0.7175	0.7182	0.7170
T + Lasso	SVM-1	0.7033	0.5093	1	0
T + Lasso	SVM-2	0.8442	0.7544	0.7000	0.8113
T + Lasso	SVM-3	0.8412	0.7589	0.6727	0.8491

AUC, area under curve; SVM-0, linear; SVM-1, polynomial; SVM-2, radial basis function kernel; SVM-3, sigmoid.

## Discussion

This study explored temporal patterns of brain function underlying SNHL and apply static and dynamic imaging radiomics features to identify SNHL biomarkers. We systematically analyzed static and dynamic imaging characteristics and applied Spearman rank correlation, LASSO, and t test plus LASSO analysis in feature selection, and then four machine learning models of SVM (linear, polynomial, RBF and sigmoid) were conducted to classify SNHL and HCs.

Machine learning models have been widely used in neuropsychiatric diseases and demonstrated that brain functional alterations had high importance of distinguish patients from controls (Gholipour et al., 2022; Qu et al., 2022). An existing study (Wasmann et al., 2022) based on peer-reviewed literature on machine learning validated the accuracy, reliability and efficiency of the automated PTA test, which is similar to manual audiometry. Machine learning models have been computed to predict hearing recovery following treatment of hearing loss (Bing et al., 2018; Koyama et al., 2021; Uhm et al., 2021). However, these studies mainly focused on clinical and hearing variables and probably ignored the influence of nonauditory symptoms. Crowson et al. used a contemporary machine learning approach to predict risk factors for depression underlying hearing loss using the Patient Health Questionnaire-9 scale (Crowson et al., 2021).

Previous imaging studies used various analysis methods to compare neural activities between SNHL patients and HCs, which were constrained by *a priori* assumptions at the group level. Our study established data-driven classifier modeling based on imaging features input by SVMs and matching these vectors with outputs, enabling us to distinguish SNHL and HCs at the individual level. SVMs are supervised learning models and can efficiently perform classification and regression (Furey et al., 2000). Using a kernel function, SVM mainly has a two-type classifier that transforms datasets into a higher-dimensional space (linear or nonlinear) (Wang et al., 2022). The linear SVM is the most common type to handle single-parameter issues. The polynomial SVM is used to process imaging data, while the RBF SVM is used when there is no prior information about the data. Moreover, the sigmoid SVM is associated with neural networks (Lee et al., 2021). Our results revealed that the RBF SVM and sigmoid SVM performed better than linear and polynomial SVM, which had relatively higher AUC and accuracy, indicating that these features were nonlinear.

Notably, three methods of feature selection were computed in our research. Taking the AUC, accuracy, sensitivity and specificity of SVM models based on selected features into consideration, we found that characteristics using Spearman rank correlation and LASSO selection had better performance. Similar to a previous study (Wang et al., 2020), we also tried a two-sample t test to explore dynamic alterations for each state, and FO (1,3,6) of the SNHL group showed significance. However, in our data-driven analysis using Spearman rank correlation, FO2 and FO4 were also included in selected features, which was not consistent with the above statistical test, indicating that the type of feature selection might have systematic inaccuracies. Therefore, in LASSO analysis, features of transition patterns between multilayer states based on sliding window and length of step were involved, shedding light on the importance of brain dynamics in the resting state.

Several auditory brain areas were selected as features in our study (Supplementary Figure S1), including the thalamus, temporal pole: superior temporal gyrus, temporal pole: middle temporal gyrus, inferior temporal gyrus, middle temporal gyrus, and superior temporal gyrus, which is consistent with reported studies (Xu et al., 2019a; Persic et al., 2020; Yang et al., 2021). Along with these auditory regions, frontal, parietal and hippocampal features selected in our study overlapped with areas in patients with hearing loss and cognitive impairments (Banaszkiewicz et al., 2021; Ponticorvo et al., 2021; Shen et al., 2021; Ma et al., 2022). Recent research has demonstrated occipital involvement in patients with auditory deprivation, suggestive of cross-modal reorganization (Campbell and Sharma, 2020). Anatomically, the angular gyrus and precuneus belong to the parietal lobule, and the calcarine fissure is part of the visual region. Micareli et al. computed positron emission tomography scanning in sudden SNHL patients, and decreased fluorodeoxyglucose uptake in the precentral, postcentral gyrus and cingulate gyrus as well as the cingulate and insula were observed (Micarelli et al., 2017), since these brain regions were linked to somatosensory and hearing function. Neuroanatomic volume differences (Yousef et al., 2021) and diffusion deficits (Moon et al., 2020) in the caudate nucleus have been detected in hearing loss and tinnitus. Interestingly, the lenticular nucleus was selected as a feature in the present study, although it has not been reported in SNHL in previous findings. The lenticular nucleus is reported as a new center regarding human motion cognitive impairments (Shan et al., 2018), and further work needs to be done to explore its potential role in SNHL.

There are some limitations in our research. First, the sample size is relatively small, and it is an early proof of data-driven analysis which needs to be repeated with a larger dataset to achieve stable efficacy. Second, this study is limited to investigating static and dynamic neural activities. Although we did not find the difference of VBM between SNHL and HCs. Further analysis of diffusion characteristics should be taken into account. Third, the present study was computed to classify SNHL and HCs, and future work could predict the risk of cognitive impairments underlying hearing loss with the combination of multiple clinical and imaging features. Moreover, the severity of hearing loss needs to be considered in further analysis. Finally, multi-connectivity topology and couplings of various states can be included in future feature selection.

In conclusion, three methods of feature selection and four types of machine learning were applied in differentiating SNHL and HCs, and Spearman rank correlation selection with RBF SVM and sigmoid SVM showed better performance. Our research might provide several promising imaging biomarkers for clinical diagnosis and contribute to a better understanding of machine learning approaches to predict the susceptibility to hearing loss.

## Data availability statement

The original contributions presented in the study are included in the article/Supplementary material, further inquiries can be directed to the corresponding authors.

## Ethics statement

The studies involving humans were approved by the Research Ethics Committee of Nanjing First Hospital. The studies were conducted in accordance with the local legislation and institutional requirements. The participants provided their written informed consent to participate in this study.

## Author contributions

YW: Conceptualization, Data curation, Formal analysis, Funding acquisition, Investigation, Writing – original draft. JY: Conceptualization, Investigation, Methodology, Software, Writing – original draft. X-MX: Conceptualization, Investigation, Software, Writing – original draft. L-LZ: Methodology, Resources, Validation, Writing – review & editing. RS: Investigation, Software, Supervision, Validation, Visualization, Writing – review & editing. SD: Methodology, Project administration, Validation, Writing – review & editing. XG: Funding acquisition, Project administration, Resources, Validation, Visualization, Writing – review & editing.

## Footnotes

^1^ http://www.nitrc.org/projects/gretna/

^2^ http://www.restfmri.net/forum/REST_V1.8
