# Supplementary material for: Combination of static and dynamic neural imaging features to distinguish sensorineural hearing loss: a machine learning study
Source: Front Neurosci. 2024 Jun 12;18:1402039. doi: 10.3389/fnins.2024.1402039 (PMC11201293; doi:10.3389/fnins.2024.1402039)
Supplement: Supplementary file 1 [file Data_Sheet_1.PDF]

Table S1. Feature selection using spearman rank correlation test in five-fold cross-validation.

K-fold	Selected Feature
Fold-1 (n=13)	FO1, ReHo69, ReHo70, ReHo57, ReHo68, ReHo62, ReHo60, ReHo16, ReHo58, ReHo15, ReHo59, FO4, ReHo61
Fold-2 (n=13)	FO1, ReHo57, ReHo60, ReHo61, ReHo70, ReHo59, ReHo69, ReHo16, ReHo10, FO2, ReHo66, ReHo62, ReHo32
Fold-3 (n=13)	FO1, ReHo32, ReHo31, ReHo15, ReHo10, ReHo61, ReHo59, ReHo60, ReHo69, ReHo57, ReHo16, BDC37, ReHo68
Fold-4 (n=13)	FO1, ReHo69, ReHo70, ReHo57, ReHo60, ReHo62, ReHo59, ReHo61, ReHo58, ReHo15, ReHo50, ReHo68, ReHo66
Fold-5 (n=13)	FO1, ReHo69, ReHo62, ReHo60, ReHo57, FO2, ReHo70, ReHo59, ReHo16, ReHo58, ReHo68, ReHo50, ReHo61

FO, fractional occupancies; ReHo, regional homogeneity; BDC, binary degree centrality. 1-90 represents different nodes in AAL90 atlas.

Table S2. Feature selection using lasso in five-fold cross-validation.

K-fold	Selected Feature
Fold-1 (n=19)	FO1, FO3, FO6, f1ALFF2, f1ALFF33, f1ALFF67, f1ALFF77, f2ALFF57, f2ALFF71, ReHo12, ReHo13, ReHo16, ReHo57, ReHo62, ReHo69, ReHo70, BDC6, BDC37, WDC66
Fold-2 (n=22)	NF 2→3_76, NF 3→3__35, NF 3→3__83, FO1, FO3, FO6, f1ALFF2, f1ALFF33, f1ALFF77, f2ALFF38, f2ALFF47, f2ALFF57, ReHo10, ReHo16, ReHo32, ReHo57, ReHo60, ReHo66, ReHo67, BDC37, BDC39, BDC69
Fold-3 (n=29)	NF 2→3_62, NF 2→3_69, NF 3→3_35, FO1, FO3, FO6, f1ALFF1, f1ALFF2, f1ALFF33, f1ALFF67, f1ALFF78, f1ALFF86, f2ALFF37, f2ALFF47, f2ALFF71, ReHo10, ReHo13, ReHo16, ReHo30, ReHo32, ReHo33, ReHo40, ReHo60, ReHo61, ReHo69, ReHo70, BDC37, BDC69, WDC24
Fold-4 (n=24)	NF 1→2_51, NF 2→3_36, NF 2→3_59, NF 3→3_35, FO1, FO3, FO6, f1ALFF2, f1ALFF67, f1ALFF77, f2ALFF1, ReHo13, ReHo29, ReHo44, ReHo57, ReHo61, ReHo66, ReHo69, ReHo70, BDC37, BDC39, BDC55, WDC23, WDC77
Fold-5 (n=56)	NF 1→2_86, NF 1→3_31, NF 1→3_86, NF 2→1_8, NF 2→1_23, NF 2→1_33, NF 2→3_2, NF 2→3_7, NF 2→3_25, NF 2→3_26, NF 2→3_36, NF 2→3_37, NF 2→3_57, NF 2→3_69, NF 2→3_76, NF 2→3_65, NF 2→3_88, NF 3→3_35, NF 3→3_81, NF 3→3_83, FO1, FO3, FO6, f1ALFF11, f1ALFF23, f1ALFF33, f1ALFF34, f1ALFF39, f1ALFF67, f1ALFF77, f1ALFF85, f2ALFF26, f2ALFF38, f2ALFF47, f2ALFF52, f2ALFF71, f2ALFF78, f2ALFF84, ReHo13, ReHo16, ReHo29, ReHo30, ReHo32, ReHo33, ReHo62, ReHo69, ReHo89, BDC37, BDC60, BDC65, BDC68, WDC15, WDC56, WDC59, WDC69

FO, fractional occupancies; NF, node flexibilities; fALFF, fractional amplitude of low frequency fluctuation; ReHo, regional homogeneity; BDC, binary degree centrality; WDC, weighted degree centrality. 1-90 represents different nodes in AAL90 atlas.

Table S3. Feature selection using t test and lasso in five-fold cross-validation.

K-fold	T-test and Lasso
Fold-1 (n=20)	NF 1→2_12, NF 1→2_13, NF 1→2_14, NF 1→2_17, NF 1→2_29, NF 1→2_40, NF 1→2_43, NF 1→2_58, NF 1→2_60, NF 1→2_69, NF 1→2_70, NF 1→2_73, NF 1→2_10, NF 1→3_13, NF 1→3_15, NF 1→3_21, NF 1→3_22, NF 1→3_23, NF 1→3_34, NF 1→3_47
Fold-2 (n=23)	NF 1→1_37, NF 1→1_76, NF 1→1_83, NF 1→1_85, NF 1→1_86, NF 1→1_87, NF 1→1_90, NF 1→2_11, NF 1→2_24, NF 1→2_35, NF 1→2_37, NF 1→2_42, NF 1→2_53, NF 1→2_59, NF 1→2_69, NF 1→2_70, NF 1→2_88, NF 1→3_1, NF 1→3_7, NF 1→3_11, NF 1→3_35, NF 1→3_36, NF 1→3_43
Fold-3 (n=33)	NF 1→1_13, NF 1→1_40, NF 1→1_81, NF 1→1_87, NF 1→2_5, NF 1→2_6, NF 1→2_8, NF 1→2_10, NF 1→2_19, NF 1→2_23, NF 1→2_27, NF 1→2_32, NF 1→2_37, NF 1→2_42, NF 1→2_45, NF 1→2_47, NF 1→2_51, NF 1→2_52, NF 1→2_62, NF 1→2_65, NF 1→2_68, NF 1→2_73, NF 1→2_78, NF 1→2_80, NF 1→2_81, NF 1→2_85, NF 1→3_11, NF 1→3_20, NF 1→3_24, NF 1→3_42, NF 1→3_47, NF 1→3_53, NF 1→3_73
Fold-4 (n=30)	NF 1→1_30, NF 1→1_45, NF 1→2_73, NF 1→2_81, NF 1→3_1, NF 1→3_5, NF 1→3_6, NF 1→3_7, NF 1→3_10, NF 1→3_18, NF 1→3_25, NF 1→3_32, NF 1→3_36, NF 1→3_43, NF 1→3_46, NF 1→3_55, NF 1→3_58, NF 1→3_70, NF 1→3_82, NF 2→1_1, NF 2→1_5, NF 2→1_13, NF 2→1_14, NF 2→1_35, NF 2→1_36, NF 2→1_38, NF 2→1_41, NF 2→1_43, NF 2→1_59, NF 2→1_74
Fold-5 (n=41)	NF 1→1_55, NF 1→1_72, NF 1→2_57, NF 1→2_62, NF 1→2_66, NF 1→3_11, NF 1→3_53, NF 1→3_55, NF 1→3_60, NF 1→3_62, NF 1→3_64, NF 1→3_69, NF 1→3_74, NF 1→3_77, NF 1→3_78, NF 1→3_79, NF 1→3_88, NF 2→1_1, NF 2→1_9, NF 2→1_13, NF 2→1_16, NF 2→1_17, NF 2→1_20, NF 2→1_21, NF 2→1_31, NF 2→1_34, NF 2→1_42, NF 2→1_43, NF 2→1_45, NF 2→1_46, NF 2→1_65, NF 2→1_72, NF 2→1_75, NF 2→1_81, NF 2→1_83, NF 2→2_5, NF 2→2_11, NF 2→2_13, NF 2→2_36, NF 2→2_37, NF 2→2_42

NF, node flexibilities. 1-90 represents different nodes in AAL90 atlas.

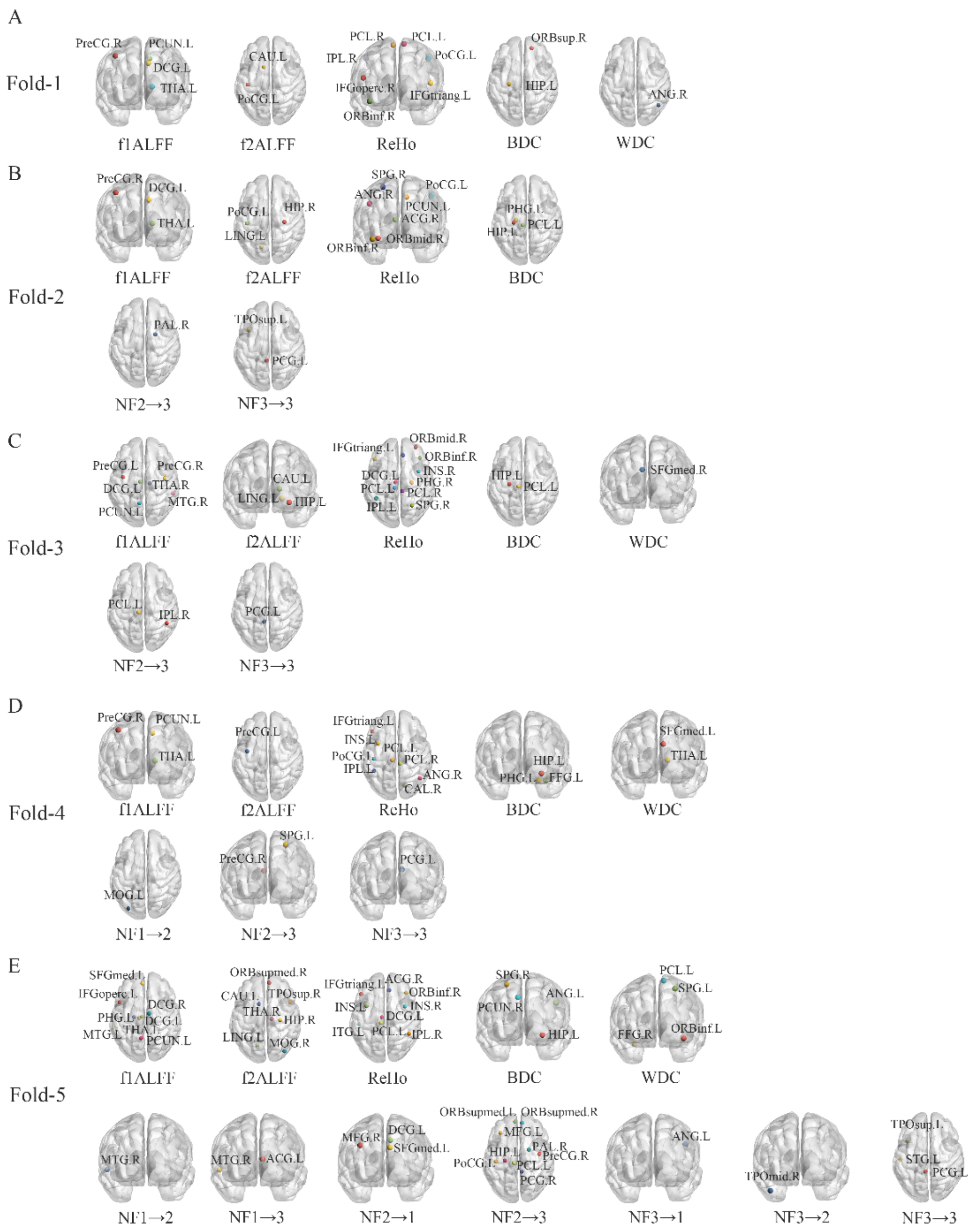

Figure S1. Selected static and dynamic features using LASSO in five-fold cross-validation. LASSO, least absolute shrinkage and selection operator; PreCG, precentral gyrus; PCUN, precuneus; DCG, median cingulate and paracingulate; THA, thalamus; CAU, caudate nucleus; PoCG, postcentral gyrus; PCL, paracentral lobule; IPL, inferior parietal lobule; IFGoperc, inferior frontal gyrus, opercular part; IFGtriang, inferior frontal gyrus, triangular part; ORBinf, inferior frontal gyrus, orbital part; ORBsup, superior frontal gyrus, orbital part; ORBmid, middle frontal gyrus, orbital part; HIP, hippocampus; ANG, angular gyrus; LING, lingual gyrus; SPG, superior parietal gyrus; ACG, anterior cingulate gyrus; PCG, posterior cingulate gyrus; PHG, parahippocampal gyrus; PAL, lenticular nucleus, pallidum; TPOsup, temporal pole: superior temporal gyrus; TPOmid, temporal pole: middle temporal gyrus; INS, insular; SFGmed, superior frontal gyrus, medial; CAL, calcarine fissure and surrounding cortex; FFG, fusiform gyrus; MOG, middle occipital gyrus; MTG, middle temporal gyrus; ORBsupmed, superior frontal gyrus, medial orbital; ITG, inferior temporal gyrus; MFG, middle frontal gyrus; STG, superior temporal gyrus;

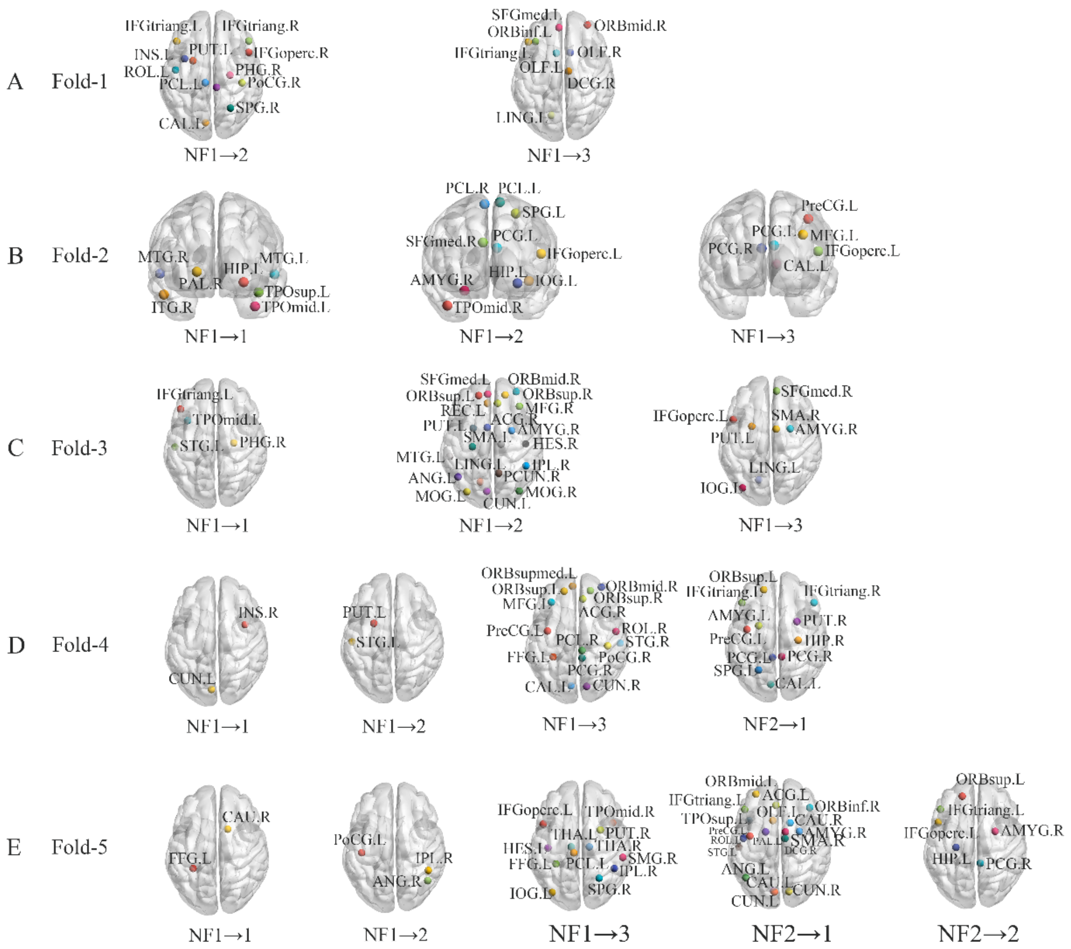

Figure S2. Selected static and dynamic features using t test as well as LASSO in five-fold cross-validation. LASSO, least absolute shrinkage and selection operator; IFGtriang, inferior frontal gyrus, triangular part; INS, insula; IFGoperc, inferior frontal gyrus, opercular part; PUT, putamen; PHG, parahippocampal gyrus; ROL, Rolandic operculum; PCL, paracentral lobule; PoCG, postcentral gyrus; SPG, superior parietal gyrus; CAL, calcarine fissure and surrounding cortex; SFGmed, superior frontal gyrus, medial; ORBinf, inferior frontal gyrus, orbital part; ORBmid, middle frontal gyrus, orbital part; ORBsup, superior frontal gyrus, orbital part; OLF, olfactory cortex; LING, lingual gyrus; MTG, middle temporal gyrus; HIP, hippocampus; PAL, lenticular nucleus, pallidum; TPOsup, temporal pole: superior temporal gyrus; TPOmid, temporal pole: middle temporal gyrus; ITG, inferior temporal gyrus; PCG, posterior cingulate gyrus; AMYG, amygdala; IOG, inferior occipital gyrus; PreCG, precentral gyrus; MFG, middle frontal gyrus; ORBsup, superior frontal gyrus, orbital part; REC, gyrus rectus; ACG, anterior cingulate gyrus; SMA, supplementary motor area; HES, Heschl gyrus; IPL, inferior parietal lobule; ANG, angular gyrus; PCUN, precuneus; MOG, middle occipital gyrus; CUN, cuneus; STG, superior temporal gyrus; FFG, fusiform gyrus; THA, thalamus; SMG, supramarginal gyrus; DCG, median cingulate and paracingulate.

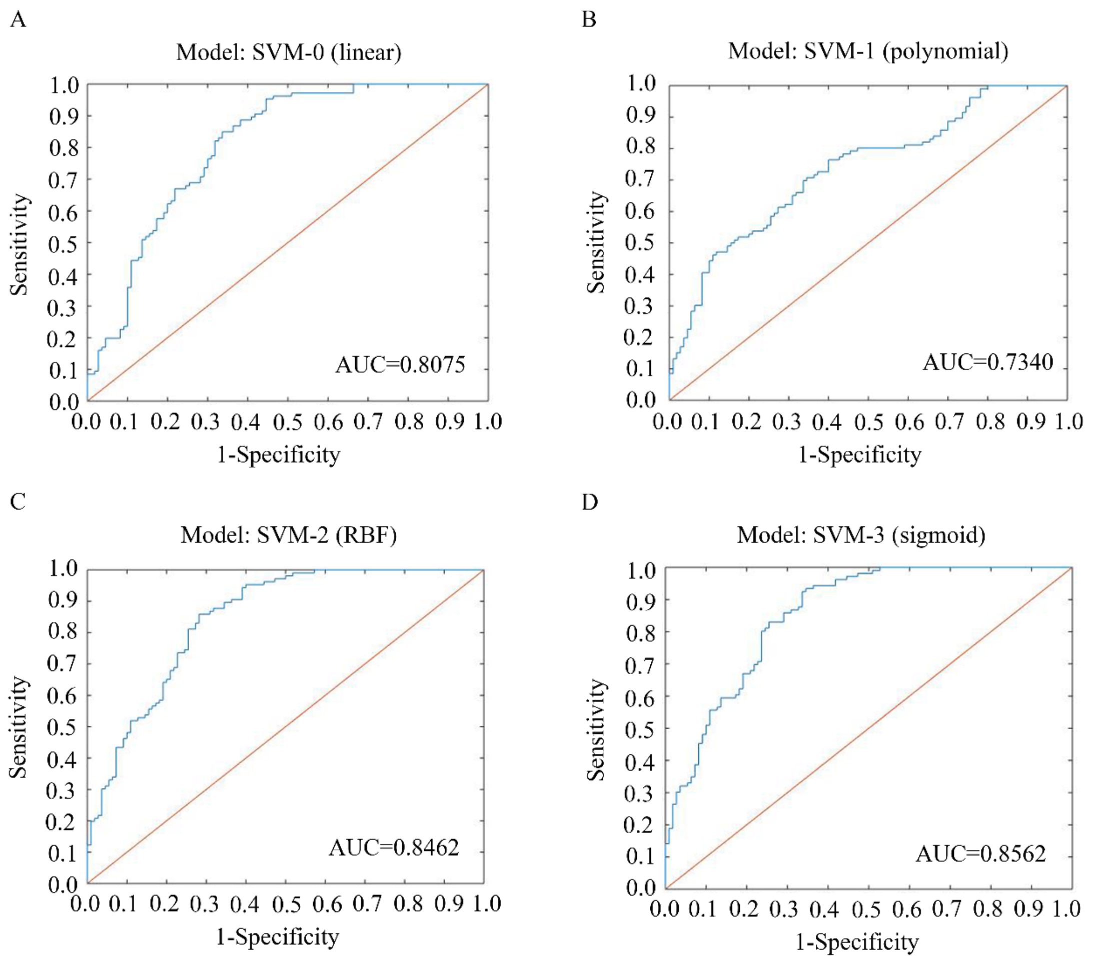

Figure S3. ROC curves and AUCs of the four SVMs using features selected by LASSO. ROC, receiver operating characteristic curve; AUC, area under the curve; SVM, support vector machine; LASSO, least absolute shrinkage and selection operator; RBF, radial basis functional kernel.

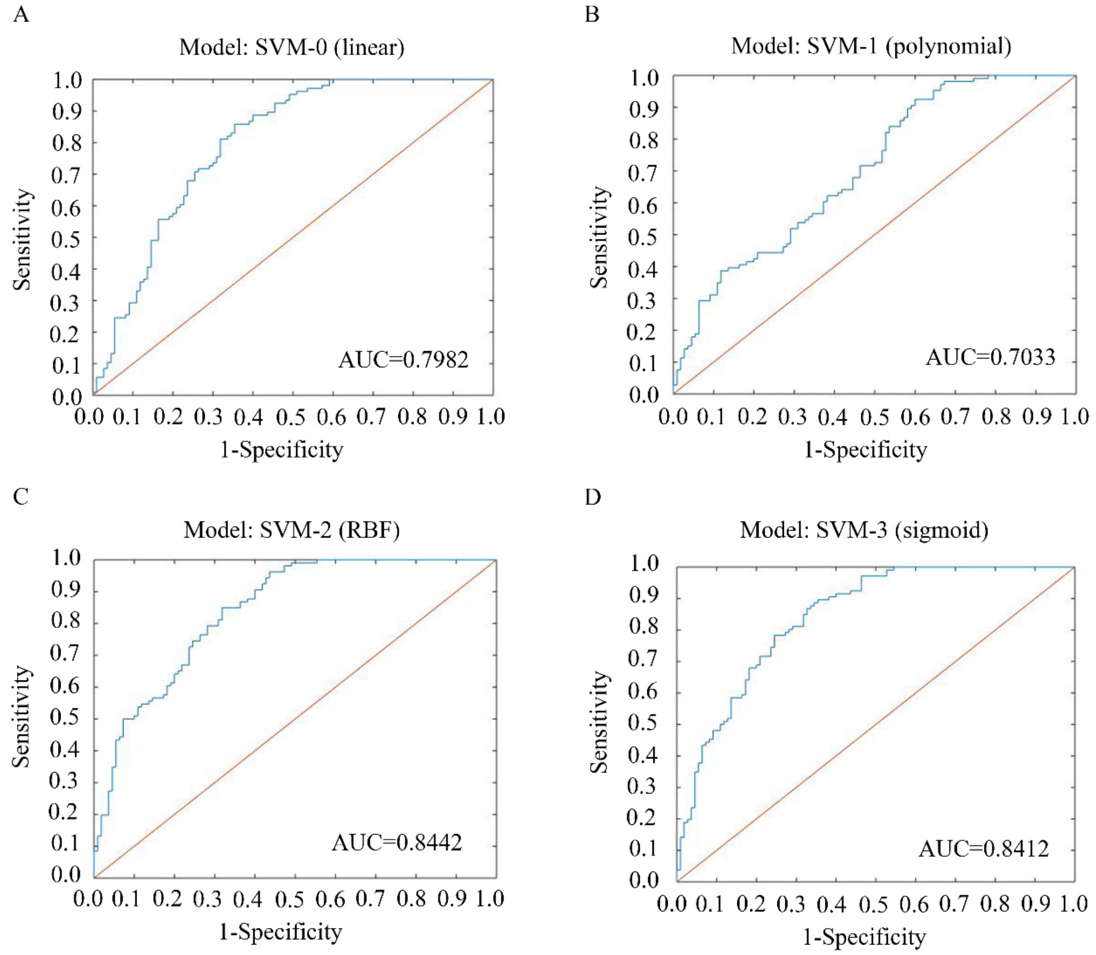

Figure S4. ROC curves and AUCs of the four SVMs using features selected by t test and LASSO. ROC, receiver operating characteristic curve; AUC, area under the curve; SVM, support vector machine; LASSO, least absolute shrinkage and selection operator; RBF, radial basis functional kernel.
